# Progression of diabetes, ischemic heart disease, and chronic kidney disease in a three chronic conditions multistate model

**DOI:** 10.1186/s12889-018-5688-y

**Published:** 2018-06-18

**Authors:** Chathura Siriwardhana, Eunjung Lim, James Davis, John J. Chen

**Affiliations:** Biostatistics Core, Department of Complementary and Integrative Medicine, University of Hawaii John A. Burns School of Medicine, Biosciences Building, Suite 211, 651 Ilalo Street, Honolulu, HI 96813 USA

**Keywords:** Aging, Chronic kidney disease, Diabetes, Ischemic heart disease, Medicare data, Multistate model

## Abstract

**Abstract:**

**Background:**

Diabetes mellitus, ischemic heart disease, and chronic kidney disease are three major chronic conditions that develop with increasing risks among adults as they get older. The interconnectedness of these three chronic conditions is well known, while each condition acts as a prognostic risk factor for the other two. It is important to understand the progressive relationships of these three conditions over time in terms of transitioning between clinical states and the impact on patients’ survival.

**Methods:**

We investigate the survival characteristics of a Medicare population aged 65 years and above in a multistate system that contained clinical states specified by death and diagnosis combinations of three chronic conditions. The study was conducted using Hawaii Medicare claims data from 2009 to 2013. To evaluate the progression of a subject with one of the newly diagnosed chronic conditions, we analyzed quantities such as state occupation probabilities in eight states and hazards of sixteen transition types. We quantified effects and significances of potential covariates such as age, gender, race/ethnicity, comorbidity burden and financial status on these temporal functions. Nonparametric method of estimating state occupation probabilities and pseudo-value based method for estimating covariate effects of a survival system were utilized.

**Results:**

We found a range of age, gender, race/ethnicity and financial status based interesting covariate influences on transitions and state occupation probabilities of the system.

**Conclusion:**

Survival characteristics of the disease system are influenced by subject-specific effects. Subgroup-specific interventions/screenings should be considered for the optimal prevention and care.

**Electronic supplementary material:**

The online version of this article (10.1186/s12889-018-5688-y) contains supplementary material, which is available to authorized users.

## Background

Diabetes mellitus (DM), ischemic heart disease (IHD), and chronic kidney disease (CKD) are three major chronic conditions that develop with increasing risks among adults as they get older [1–3]. In recent years, approximately 31.0, 25.9, and 4.4% of the elderly US population aged 65 years and above suffered from DM, IHD, and CKD, respectively. These three chronic conditions are associated with high health care expenses [4–6]. In 2013, the total health care costs for DM, IHD, and CKD were 101.4, 88.1, and 13.5 billion dollars, and the elderly population accounted for 42.8, 61.2, 52.5% of these costs, respectively [7].

The interconnectedness of these three chronic conditions is well known, while each condition acts as a prognostic risk factor for the other two. Previous studies discuss risks associated between pairs of these conditions and their subtypes on lifetime, quality of life, and etiological aspects. For example, a diagnosis of CKD not only increases the risk of cardiovascular morbidity but worsens cardiovascular condition outcomes if a subject has both diseases together [8]. Some evidence suggests that around half of all heart failure patients suffer at least some level of CKD in their lifetime [9]. Accordingly, patients with CKD are strongly recommended to have regular screenings for the presence of cardiovascular related complications for early intervention. Cardiovascular complications also play a critical role in the development of CKD [10, 11], and evidence shows that CKD prevalence is high among the population with undiagnosed diabetes and pre-diabetes [12]. In addition, DM is reported as a major risk factor for cardiovascular disease [13] and a leading risk factor for end-stage renal disease, co-occurring in half of the patients with CKD [14, 15].

Generally, these three conditions do not necessarily follow a generic progression pattern, but one condition or pairs of them act as a possible risk factor for developing the other conditions. Therefore, it is important to understand the progressive relationships of these three conditions over time in terms of transitioning between clinical states and the impact on patients’ survival, especially among the elderly, who are at increased risk for these conditions. To our knowledge, there is no published literature discussing these conditions together with respect to state transitioning and patients’ survival. To fill this gap, we investigated the characteristics of a progressive multistate system structured with these three major chronic conditions for elderly individuals using Hawaii Medicare data.

The main objectives of this study are two-fold: (1) to determine overall survival characteristics associated with DM, IHD, and CKD conditions in adults aged 65 years and above as related to transitions between various clinical stages; and (2) to demonstrate the use of health insurance data to evaluate the survival characteristics of a chronic disease system.

## Methods

### Data

We employed a retrospective cohort analysis of 23,030 individuals aged 65 years or above who experienced at least one of the following conditions: DM, IHD, and CKD, from January 1, 2009 through December 31, 2013 using Hawaii Medicare claims data. This data allowed us to systematically track the occurrence of a set of selected disease conditions for up to five years and provided covariate information such as demographic data and the presence of other co-morbidities. Data on both inpatient and outpatient visits were utilized. The International Classification of Disease 9th Revision (ICD-9) diagnosis codes [16] were used for specifying the disease conditions. Specifically, the series of ICD-9 codes of 250, 410–414, and 585 were used to identify DM, IHD, and CKD, respectively.

The Medicare master beneficiary summary file was used to identify gender and race/ethnicity. Race/ethnicity was categorized as White, Asian, Native Hawaiian and Pacific Islander (NHPI), and Other group, which contained American Indian/Alaska Native, African-American, Hispanic and unknown races that represented as a small proportion. Respective percentages of these groups were White, 28.4%; Asian, 26.9%; NHPI, 24.0%; and Other, 20.7%. In addition, we used the Charlson Comorbidity Index (CCI) calculated at baseline that gives a one-year survival score for a patient based on chronic commodities [17]. Furthermore, we used the dual eligibility status of an individual who received both Medicare and Medicaid benefits as an indicator for socioeconomic status as it is common among individuals with low incomes. The research protocol was approved by the Institutional Review Board at the University of Hawaii.

### Multistate model and statistical analyses

#### Multistate model

**Fig. 1:**
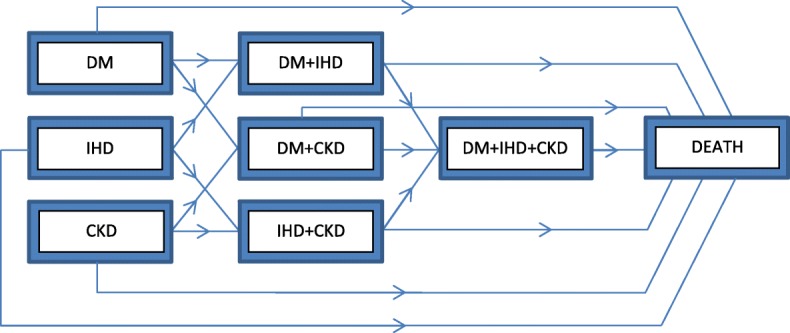
Graphical representation of the progressive chronic disease network consisting of eight states

In this study, we used the acyclic multistate model shown in Fig. 1 to define an interconnected progressive chronic disease system for the elderly population. In the system, there are eight clinical states an individual can occupy at a given time point. An individual starts from one of the single disease states (i.e., DM, IHD, or CKD) and moves towards the absorbing state Death either directly or through four different intermediate multi-disease states. Depending on the initial state of the individual, he/she may move to one of three dyad states: DM + IHD, DM + CKD, IHD + CKD, or to the Death state. If an individual moves to one of the dyad states, he/she may transfer to DM + IHD + CKD, or to the Death state. We controlled the complexity of the system by limiting the initial states of an individual to be one of DM, IHD, or CKD only, and by not allowing direct transitions from states DM, IHD, and CKD directly to the DM + IHD + CKD state.

We determined the earliest date of the first disease occurrence for each individual and tracked all subsequent states along with state transition times. Starting from this point, we extracted the claims data for the three conditions to track the progression of each individual in various clinical states as indicated in Fig. 1. Here, individuals entered the system at different time points, resulting in right-censoring in the multistate model. A summary of transition counts for the system is provided in Additional file 1: Table S1 in matrix form.

### State occupation probabilities and transition hazards

State occupation probabilities and state-to-state transition hazards are two important quantities for a multistate model. We used the Aalen and Johansen [18] estimator to estimate marginal state occupational probabilities, which is a nonparametric technique that provides great flexibility in handling a complex multistate system without strict model assumptions. To identify the important covariates affecting state occupation probabilities and state-to-state transition hazards, we employed the pseudo-values methods proposed by Andersen and Klein [19]. This was implemented using the generalized linear model with a *logit* link function to estimate the covariate effect on the state occupation while incorporating *log* link function for state-to-state cumulative transition hazards. Details of the aforementioned estimation procedures for a multistate model are described in the Additional file 1.

Due to the limited follow-up period the observed transitions subjected to right-censoring. The estimation was based on the assumption that individuals were randomly censored. Ninety-five percent point-wise confidence intervals of state occupational probabilities and state-to-state transition hazards were estimated using the bootstrap method with 1000 bootstrap samples. All the analyses were conducted using R-3.3.1 software, with algorithms developed incorporating existing functions in R-base packages.

## Results

We evaluated the progression of a newly diagnosed subject with DM, IHD, and CKD conditions with respect to time, analyzing the state occupation probabilities of eight different states, and the hazards of sixteen transition types (see Fig. 1). We also reported effects of several important covariates, such as age, gender, race/ethnicity, comorbidity burden and financial status, on these temporal functions.

### Transition counts

At baseline, individuals entered the system initiating from 9628 DM, 7943 IHD, and 5359 CKD states. Correspondingly, 6868 (71.3%), 5276 (66.4%), and 3295 (61.4%) of these individuals remained in their initial states by the end of the study period. We summarized the transition counts for the study in Additional file 1: Table S1 to illustrate the basic transition characteristics for the system. For example, the cell (DM, DM + IHD) shows that of the 9628 who initiated from DM state, 1163 individuals transferred to DM + IHD state. The cell (DM + IHD + CKD, Death) indicates that out of 950 individuals who transferred to DM + IHD + CKD state, 363 individuals transferred to Death state.

### Marginal estimates of state occupation probability

**Fig. 2:**
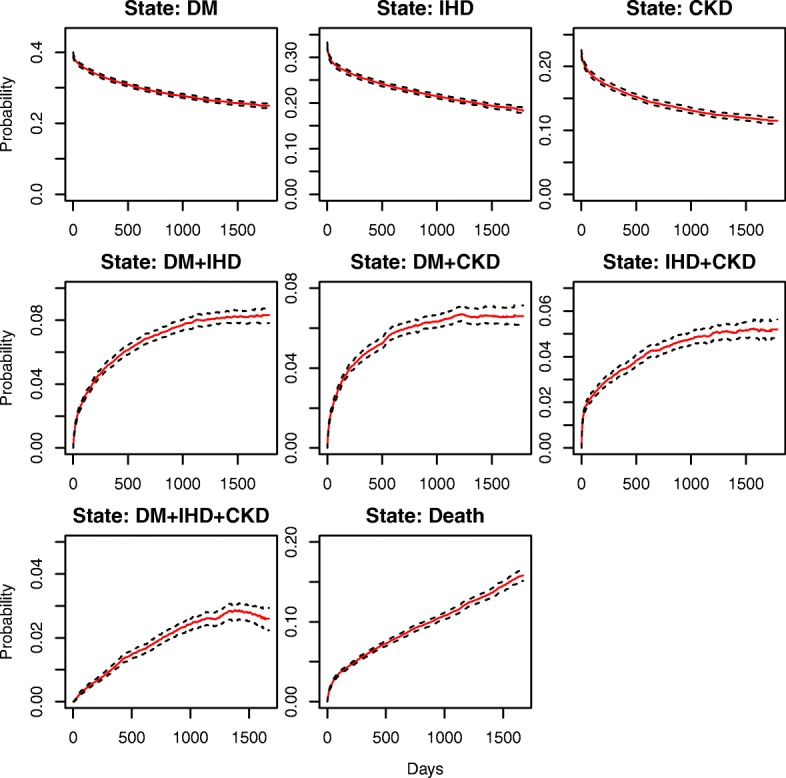
Estimated marginal state occupational probabilities for the multistate model along with 95% bootstrap based confidence bands. We illustrate state occupation probabilities with a solid line and corresponding 95% confidence bands with dotted lines. Note: different spans of y axis were used for different sets of states

The initial probabilities of entering the system from DM, IHD, and, CKD clinical states were 41.9, 34.7, and 23.4%. The probabilities of staying in these states gradually declined over time down to 27.3, 20.6, and 12.7% four years later. CKD showed the highest relative decline in the state occupational probability, resulting in the tail probability closer to half of its initial value. In Fig. 2, we provided a graphical illustration of the marginal state occupation probabilities that estimated via the Aalen-Johansen estimator along with 95% bootstrap based point-wise confidence intervals.

After the initial state, the next three subsequent intermediate states are three dyads: DM + IHD, DM + CKD, and IHD + CKD. For these dyad sets, the state occupation probabilities increase over time with a nonlinear trend, such that they increase rapidly during the initial phase. At a later phase the degree of increment slowly dissipates, resulting in approximately 8.7, 7.0, and 5.7% for occupying DM + IHD, DM + CKD, and IHD + CKD after four years of the initial occurrence, respectively.

The occupation probability of the DM + IHD + CKD state increases at a much slower rate compared to the dyad state sets reaching a maximum value of 3.0% around 1415 days, and then declining at a very slow pace. Clearly, the intensities of state occupation probabilities for intermediate states at any given time are relatively lower than those of the initial states.

For the absorbing state of Death, the occupation probability increases rapidly at the beginning and then follows an almost linear trend with respect to time. After four years, the probability of experiencing death reaches approximately 15.1%.

### Marginal estimates of cumulative transition hazards

**Fig. 3:**
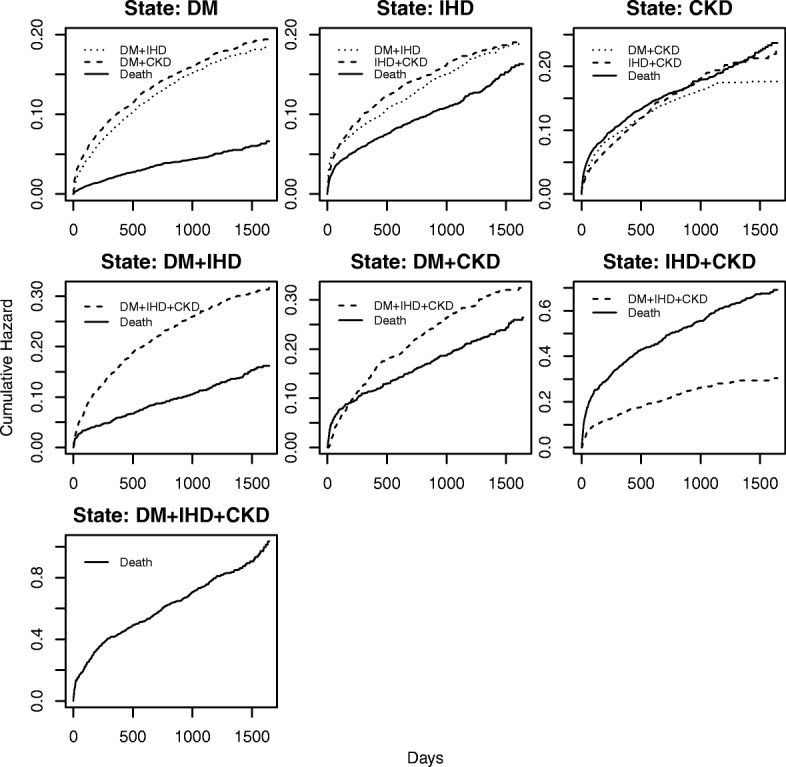
Estimated marginal cumulative state-to-state transition hazards for the multistate model. Note: different spans of y-axis were used for different states

Figure 3 presents the marginally estimated state-to-state cumulative transition hazards from one given state to another by the Nelson-Aalen type hazard estimator. The details of 95% bootstrap based point-wise confidence intervals of the estimated state-to-state cumulative transition can be found in Additional file 1: Figures S1-S7.

Among all the initial single disease states, subjects with DM were least likely to die at any given time. On the other hand, subjects with CKD had the highest risk of death. The corresponding death risk for CKD subjects appears to be similar to their transition hazards to any subsequent dyad states. For a subject with IHD, the risk of death rapidly increased but at a slower rate compared to a subject with CKD.

Compared to other dyad states, the DM + IHD had the lowest risk of death at any given time, followed by DM + CKD. Subjects with IHD + CKD had a very high cumulative hazard of death, which was about six times higher compared with the corresponding values of the DM + IHD state at the 4th year. Furthermore, the hazard of death at IHD + CKD was far higher than the hazard of transitioning to DM + IHD + CKD state, showing that these individuals were less likely to move to the triad state. As noted, there was an elevated risk of death for DM + CKD subjects during the very early period compared with transitioning to DM + IHD + CKD. For the entire system, we observed the highest risk of death by DM + IHD + CKD at any given time.

### Covariate effects on state occupation probabilities

Table 1 presents the estimated odds ratios and 95% confidence bands on state occupations for several covariates of interest such as age, gender, race/ethnicity, CCI, and dual eligibility. Note that a multistate system is characterized by state-to-state events and at-risk processes, which are both functions of time. Consequently, the covariate effect on the state occupation could vary over time. Therefore, we report the impact of covariate at a sequence of time points: at 365, 730, 1095, and 1460 days corresponding to years 1, 2, 3, and 4. The estimated regression coefficients from the multistate model along with *p*-values are presented in Additional file 1: Table S2.

**Table 1:** Estimated odds ratios of state occupation along with 95% confidence bands at several time points for a set of covariates given by age, gender, race/ethnicity, CCI and dual eligibility. For age and CCI variables, the odds ratio is defined as the ratio of odds for a unit increment of the variable. For gender, race/ethnicity and dual eligibility the ratio is calculated relative to males, whites, and ineligibility, respectively

State	Days	Age	Gender (Female vs. Males)	Race/Ethnicity			CCI	Dual Eligibility
				Asian vs. White	NHPI vs. White	Others vs. White		
DM	0	0.960 (0.957, 0.963)	1.431 (1.356, 1.511)	1.713 (1.589, 1.847)	1.890 (1.753, 2.038)	1.745 (1.613, 1.886)	0.782 (0.759, 0.805)	0.931 (0.862, 1.005)
	365	0.962 (0.960, 0.965)	1.550 (1.464, 1.640)	1.636 (1.512, 1.769)	1.867 (1.725, 2.020)	1.653 (1.523, 1.794)	0.754 (0.728, 0.781)	0.892 (0.823, 0.966)
	730	0.962 (0.959, 0.965)	1.582 (1.493, 1.676)	1.599 (1.476, 1.733)	1.857 (1.714, 2.013)	1.634 (1.503, 1.777)	0.752 (0.725, 0.780)	0.863 (0.795, 0.936)
	1095	0.961 (0.959, 0.964)	1.603 (1.512, 1.700)	1.571 (1.449, 1.704)	1.816 (1.675, 1.970)	1.605 (1.475, 1.747)	0.753 (0.725, 0.781)	0.860 (0.792, 0.934)
	1460	0.960 (0.958, 0.963)	1.601 (1.510, 1.698)	1.577 (1.453, 1.712)	1.837 (1.693, 1.993)	1.623 (1.491, 1.766)	0.755 (0.728, 0.784)	0.848 (0.781, 0.922)
IHD	0	1.027 (1.024, 1.030)	0.833 (0.788, 0.881)	0.506 (0.470, 0.545)	0.572 (0.531, 0.617)	0.529 (0.488, 0.572)	1.045 (1.024, 1.067)	1.067 (0.986, 1.155)
	365	1.018 (1.015, 1.021)	0.952 (0.897, 1.010)	0.504 (0.466, 0.545)	0.558 (0.515, 0.604)	0.499 (0.459, 0.544)	1.003 (0.981, 1.026)	0.971 (0.891, 1.057)
	730	1.015 (1.012, 1.018)	0.968 (0.911, 1.028)	0.505 (0.465, 0.547)	0.566 (0.522, 0.613)	0.508 (0.466, 0.554)	0.996 (0.973, 1.019)	0.926 (0.848, 1.011)
	1095	1.013 (1.010, 1.016)	0.983 (0.924, 1.045)	0.499 (0.460, 0.541)	0.566 (0.521, 0.614)	0.504 (0.462, 0.551)	0.997 (0.974, 1.021)	0.888 (0.811, 0.971)
	1460	1.012 (1.008, 1.015)	0.996 (0.936, 1.060)	0.499 (0.459, 0.541)	0.568 (0.523, 0.617)	0.499 (0.457, 0.546)	0.998 (0.974, 1.022)	0.878 (0.802, 0.962)
CKD	0	1.023 (1.020, 1.026)	0.996 (0.936, 1.061)	1.007 (0.927, 1.095)	0.865 (0.793, 0.944)	0.957 (0.875, 1.047)	1.198 (1.173, 1.223)	1.067 (0.977, 1.164)
	365	1.019 (1.016, 1.023)	1.126 (1.051, 1.207)	0.926 (0.845, 1.014)	0.832 (0.756, 0.916)	0.895 (0.810, 0.988)	1.070 (1.046, 1.096)	1.010 (0.916, 1.114)
	730	1.017 (1.013, 1.020)	1.153 (1.072, 1.239)	0.909 (0.826, 1.000)	0.839 (0.760, 0.927)	0.881 (0.795, 0.977)	1.056 (1.030, 1.082)	0.981 (0.885, 1.087)
	1095	1.015 (1.011, 1.019)	1.134 (1.053, 1.220)	0.900 (0.817, 0.992)	0.848 (0.766, 0.938)	0.881 (0.793, 0.979)	1.057 (1.031, 1.084)	0.983 (0.885, 1.091)
	1460	1.013 (1.010, 1.017)	1.138 (1.057, 1.226)	0.904 (0.820, 0.997)	0.836 (0.755, 0.925)	0.881 (0.793, 0.980)	1.061 (1.035, 1.088)	0.959 (0.863, 1.066)
DM + IHD	365	0.991 (0.986, 0.997)	0.690 (0.612, 0.777)	0.943 (0.800, 1.110)	1.166 (0.995, 1.366)	1.114 (0.944, 1.314)	0.795 (0.735, 0.860)	1.154 (0.982, 1.356)
	730	0.990 (0.985, 0.995)	0.736 (0.661, 0.819)	0.981 (0.846, 1.137)	1.160 (1.004, 1.340)	1.099 (0.945, 1.278)	0.801 (0.747, 0.859)	1.066 (0.918, 1.237)
	1095	0.991 (0.986, 0.996)	0.743 (0.670, 0.825)	0.978 (0.847, 1.129)	1.210 (1.052, 1.391)	1.116 (0.964, 1.293)	0.797 (0.744, 0.853)	1.100 (0.953, 1.271)
	1460	0.990 (0.985, 0.995)	0.779 (0.703, 0.864)	0.976 (0.846, 1.126)	1.192 (1.038, 1.370)	1.081 (0.933, 1.252)	0.792 (0.739, 0.849)	1.060 (0.918, 1.225)
DM + CKD	365	0.975 (0.969, 0.980)	0.916 (0.809, 1.037)	1.794 (1.498, 2.148)	1.527 (1.265, 1.843)	1.903 (1.583, 2.288)	0.827 (0.764, 0.895)	0.874 (0.733, 1.042)
	730	0.975 (0.970, 0.980)	1.017 (0.907, 1.141)	1.719 (1.457, 2.027)	1.528 (1.288, 1.813)	1.717 (1.448, 2.037)	0.810 (0.750, 0.874)	0.866 (0.735, 1.019)
	1095	0.976 (0.971, 0.981)	1.069 (0.955, 1.196)	1.736 (1.476, 2.043)	1.562 (1.320, 1.848)	1.745 (1.475, 2.064)	0.810 (0.752, 0.873)	0.868 (0.740, 1.019)
	1460	0.976 (0.971, 0.981)	1.001 (0.898, 1.117)	1.803 (1.540, 2.111)	1.607 (1.365, 1.891)	1.813 (1.540, 2.134)	0.799 (0.742, 0.860)	0.914 (0.784, 1.065)
HD + CKD	365	1.042 (1.034, 1.049)	0.654 (0.564, 0.758)	0.809 (0.670, 0.978)	0.728 (0.594, 0.892)	0.846 (0.687, 1.041)	1.017 (0.965, 1.071)	1.331 (1.095, 1.619)
	730	1.037 (1.031, 1.044)	0.757 (0.662, 0.864)	0.758 (0.637, 0.902)	0.758 (0.633, 0.909)	0.792 (0.655, 0.957)	1.007 (0.959, 1.057)	1.236 (1.031, 1.483)
	1095	1.034 (1.027, 1.040)	0.768 (0.676, 0.872)	0.755 (0.640, 0.891)	0.744 (0.626, 0.885)	0.815 (0.681, 0.975)	1.003 (0.957, 1.051)	1.133 (0.949, 1.353)
	1460	1.033 (1.027, 1.040)	0.786 (0.693, 0.893)	0.744 (0.630, 0.878)	0.732 (0.616, 0.870)	0.816 (0.683, 0.975)	0.984 (0.936, 1.034)	1.143 (0.959, 1.364)
DM + IHD + CKD	365	0.977 (0.968, 0.986)	0.513 (0.409, 0.643)	1.784 (1.323, 2.407)	1.460 (1.063, 2.005)	1.496 (1.085, 2.063)	0.862 (0.761, 0.976)	1.207 (0.911, 1.599)
	730	0.980 (0.973, 0.987)	0.528 (0.442, 0.630)	1.729 (1.361, 2.197)	1.316 (1.016, 1.706)	1.852 (1.450, 2.365)	0.846 (0.764, 0.936)	1.347 (1.085, 1.673)
	1095	0.979 (0.973, 0.986)	0.521 (0.442, 0.613)	1.792 (1.437, 2.235)	1.414 (1.118, 1.789)	1.739 (1.382, 2.188)	0.785 (0.703, 0.877)	1.172 (0.952, 1.442)
	1460	0.980 (0.974, 0.987)	0.524 (0.446, 0.616)	1.665 (1.336, 2.075)	1.368 (1.084, 1.725)	1.859 (1.488, 2.322)	0.751 (0.667, 0.846)	1.102 (0.894, 1.358)
Death	365	1.081 (1.075, 1.088)	0.654 (0.584, 0.733)	0.869 (0.751, 1.006)	0.816 (0.696, 0.957)	0.932 (0.790, 1.101)	1.477 (1.441, 1.514)	1.485 (1.279, 1.723)
	730	1.078 (1.072, 1.083)	0.632 (0.573, 0.697)	0.883 (0.780, 1.000)	0.747 (0.651, 0.857)	0.888 (0.771, 1.022)	1.450 (1.416, 1.485)	1.631 (1.438, 1.851)
	1095	1.077 (1.072, 1.081)	0.658 (0.601, 0.720)	0.899 (0.801, 1.009)	0.755 (0.665, 0.858)	0.909 (0.798, 1.036)	1.417 (1.385, 1.451)	1.695 (1.508, 1.905)
	1460	1.077 (1.072, 1.081)	0.653 (0.599, 0.712)	0.924 (0.827, 1.033)	0.806 (0.714, 0.910)	0.937 (0.827, 1.062)	1.394 (1.362, 1.427)	1.784 (1.597, 1.994)

Age significantly affected the state occupation probabilities of all eight states and its effect was fairly uniform throughout the four time points for a given state, except for IHD and CKD. Interestingly, the direction of age effect appeared to be inconsistent among states. For example, older individuals had low odds of occupying the DM state or dyads and triad states with DM at any time. As expected, the odds of occupying the Death state for an older individual was relatively higher and the magnitude of effects due to age increment appeared to be the highest among all states.

Gender showed significant impacts on all states except IHD and DM + CKD. Clearly, the odds of occupying DM and CKD states for females were higher than males at any given time and the corresponding impact was relatively large for DM state. On the other hand, males had higher odds to be in DM + IHD, IHD + CKD, DM + IHD + CKD, and Death states at all the time points studied.

We evaluated the race/ethnicity difference on the state occupation probability, estimating odds ratios for other racial/ethnic groups, relative to White. The result suggested a fair consistency in racial/ethnic effects on the state occupation probability among Asian, NHPI and Other. Compared to Whites, for example, Asian, NHPI and Other had significantly higher odds to occupy DM, DM + CKD, DM + IHD + CKD states but low odds to be in IHD and IHD + CKD states at all four time points. Additionally, for NHPIs, there was a significantly high odds to be in DM + IHD and a low odds to be in Death state, compared to Whites.

The CCI appeared to be another important factor for state occupation in this multistate model. Higher values of CCI were associated with significantly higher odds for an individual to occupy the Death state at any given time. Subjects with higher CCI values were less likely to stay in DM, DM + IHD, DM + CKD, and DM + IHD + CKD states, compared to subjects with lower values. On the other hand, individuals with high CCI had higher odds to occupy the CKD state. Our results did not suggest any statistically significant associations between CCI and odds to be in IHD and IHD + CKD states.

Interestingly, the dual eligibility status did not show strong effects on the state occupation probabilities of the system except for DM and Death states. The dual eligibility factor reduced the odds to be in the DM state. However, an individual with the dual eligibility had significantly higher odds of occupying the Death state compared to non-eligible subjects, and the intensity of the effect increased over time.

### Covariate effects on cumulative transition hazards

Table 2 summarizes the estimated hazard ratios for the 16 different cumulative state-to-state transitions and their corresponding 95% point-wise confidence bands for the set of covariates (See Additional file 1: Table S3 for regression coefficients). The “From” and “To” columns of the table represent the type of transition and the rest of the columns provide relative cumulative hazards corresponding to each covariate. For example, the first cell under age variable shows that the relative cumulative transition hazard from DM to DM + IHD was about 0.994 after one year due to a unit increment in age. Here, we focus our discussion comparing cumulative hazards at the beginning of the 4th year.

**Table 2:** Estimated cumulative transition hazard ratios along with 95% confidence bands at elected time points for a set of covariates given by age, gender, race/ethnicity, CCI and dual eligibility. For age and CCI, the odds ratio is defined as the ratio of odds for a unit increment of the variable. For gender, race/ethnicity and dual eligibility the ratio is calculated relative to males, whites, and ineligibility, respectively

States		Days	Age	Gender (Females vs. Males)	Race/Ethnicity			CCI	Dual Eligibility
From	To				Asian vs. White	NHPI vs. white	Others vs. White		
DM	DM + IHD	365	0.994 (0.990, 0.997)	0.863 (0.799, 0.932)	0.971 (0.875, 1.077)	1.009 (0.907, 1.122)	1.014 (0.908, 1.132)	0.940 (0.912, 0.968)	1.183 (1.061, 1.319)
		730	0.996 (0.993, 0.999)	0.849 (0.793, 0.910)	1.010 (0.920, 1.108)	1.041 (0.947, 1.145)	1.073 (0.972, 1.185)	0.936 (0.912, 0.961)	1.138 (1.032, 1.254)
		1095	0.997 (0.994, 1.000)	0.848 (0.793, 0.908)	1.055 (0.963, 1.155)	1.122 (1.022, 1.231)	1.111 (1.008, 1.224)	0.934 (0.910, 0.958)	1.082 (0.983, 1.190)
		1460	0.999 (0.995, 1.002)	0.904 (0.842, 0.971)	1.029 (0.935, 1.132)	1.062 (0.963, 1.171)	1.074 (0.970, 1.189)	0.927 (0.902, 0.952)	1.088 (0.984, 1.203)
DM	DM + CKD	365	0.989 (0.986, 0.992)	0.944 (0.879, 1.013)	1.258 (1.143, 1.384)	1.156 (1.049, 1.274)	1.275 (1.152, 1.411)	0.941 (0.916, 0.967)	0.974 (0.882, 1.076)
		730	0.990 (0.987, 0.993)	0.978 (0.916, 1.043)	1.241 (1.138, 1.354)	1.143 (1.046, 1.249)	1.214 (1.107, 1.332)	0.938 (0.915, 0.961)	0.996 (0.909, 1.091)
		1095	0.991 (0.988, 0.994)	0.972 (0.911, 1.037)	1.241 (1.137, 1.353)	1.160 (1.062, 1.268)	1.219 (1.111, 1.337)	0.942 (0.919, 0.965)	0.992 (0.905, 1.086)
		1460	0.992 (0.989, 0.996)	0.951 (0.888, 1.018)	1.234 (1.125, 1.354)	1.161 (1.056, 1.276)	1.217 (1.103, 1.342)	0.947 (0.922, 0.972)	1.042 (0.946, 1.148)
IHD	DM + IHD	365	0.996 (0.992, 1.000)	0.750 (0.694, 0.810)	1.021 (0.920, 1.134)	1.167 (1.049, 1.299)	1.102 (0.986, 1.232)	0.956 (0.928, 0.984)	1.064 (0.953, 1.187)
		730	0.995 (0.992, 0.999)	0.802 (0.747, 0.862)	1.056 (0.959, 1.163)	1.140 (1.033, 1.258)	1.073 (0.968, 1.189)	0.960 (0.934, 0.987)	1.050 (0.949, 1.162)
		1095	0.996 (0.993, 0.999)	0.805 (0.749, 0.864)	1.075 (0.977, 1.184)	1.142 (1.035, 1.259)	1.046 (0.945, 1.159)	0.956 (0.930, 0.982)	1.111 (1.005, 1.228)
		1460	0.997 (0.994, 1.001)	0.789 (0.732, 0.850)	1.085 (0.982, 1.199)	1.150 (1.038, 1.274)	1.087 (0.977, 1.209)	0.949 (0.922, 0.976)	1.111 (1.000, 1.234)
IHD	IHD + CKD	365	1.022 (1.018, 1.026)	0.797 (0.736, 0.863)	0.970 (0.871, 1.079)	0.908 (0.814, 1.013)	0.999 (0.891, 1.119)	1.056 (1.024, 1.088)	1.234 (1.103, 1.380)
		730	1.022 (1.019, 1.026)	0.813 (0.755, 0.875)	0.924 (0.836, 1.020)	0.884 (0.799, 0.978)	0.964 (0.867, 1.071)	1.039 (1.010, 1.069)	1.239 (1.117, 1.374)
		1095	1.022 (1.019, 1.026)	0.813 (0.757, 0.875)	0.951 (0.863, 1.049)	0.890 (0.805, 0.983)	1.004 (0.905, 1.114)	1.028 (1.000, 1.057)	1.166 (1.052, 1.291)
		1460	1.022 (1.018, 1.025)	0.807 (0.748, 0.871)	0.922 (0.832, 1.022)	0.864 (0.778, 0.960)	1.012 (0.907, 1.129)	1.018 (0.988, 1.048)	1.131 (1.016, 1.260)
CKD	DM + CKD	365	0.987 (0.982, 0.991)	0.876 (0.797, 0.963)	1.420 (1.251, 1.612)	1.217 (1.069, 1.385)	1.404 (1.227, 1.606)	0.993 (0.957, 1.029)	0.971 (0.850, 1.109)
		730	0.989 (0.985, 0.993)	0.911 (0.835, 0.993)	1.415 (1.259, 1.591)	1.230 (1.092, 1.386)	1.425 (1.258, 1.613)	0.983 (0.951, 1.016)	0.942 (0.834, 1.064)
		1095	0.989 (0.985, 0.993)	0.941 (0.862, 1.027)	1.402 (1.246, 1.576)	1.211 (1.074, 1.365)	1.389 (1.226, 1.574)	0.964 (0.932, 0.997)	0.911 (0.806, 1.031)
		1460	0.990 (0.986, 0.994)	0.920 (0.841, 1.006)	1.390 (1.232, 1.567)	1.254 (1.109, 1.418)	1.404 (1.236, 1.596)	0.962 (0.929, 0.995)	0.916 (0.808, 1.038)
CKD	IHD + CKD	365	1.014 (1.009, 1.018)	0.832 (0.755, 0.917)	0.972 (0.853, 1.109)	0.879 (0.768, 1.005)	0.954 (0.829, 1.096)	1.052 (1.014, 1.092)	1.131 (0.986, 1.298)
		730	1.013 (1.009, 1.017)	0.880 (0.807, 0.959)	0.987 (0.879, 1.108)	0.893 (0.793, 1.005)	0.944 (0.835, 1.068)	1.039 (1.005, 1.074)	1.101 (0.975, 1.242)
		1095	1.013 (1.009, 1.017)	0.938 (0.861, 1.021)	0.990 (0.883, 1.110)	0.860 (0.765, 0.966)	0.917 (0.813, 1.036)	1.037 (1.004, 1.071)	1.046 (0.928, 1.178)
		1460	1.014 (1.010, 1.019)	0.960 (0.878, 1.050)	0.964 (0.855, 1.087)	0.900 (0.796, 1.018)	0.936 (0.823, 1.063)	1.023 (0.989, 1.059)	1.111 (0.980, 1.260)
DM + IHD	DM + IHD + CKD	365	1.000 (0.993, 1.006)	0.674 (0.583, 0.779)	0.922 (0.758, 1.121)	0.974 (0.798, 1.190)	1.036 (0.841, 1.275)	0.970 (0.917, 1.025)	1.353 (1.103, 1.660)
		730	0.998 (0.992, 1.003)	0.713 (0.634, 0.802)	1.002 (0.856, 1.173)	1.007 (0.857, 1.183)	1.129 (0.954, 1.335)	0.961 (0.919, 1.005)	1.354 (1.148, 1.597)
		1095	0.996 (0.991, 1.001)	0.737 (0.663, 0.820)	1.078 (0.934, 1.244)	1.067 (0.922, 1.235)	1.136 (0.976, 1.323)	0.954 (0.916, 0.994)	1.241 (1.068, 1.441)
		1460	0.999 (0.994, 1.004)	0.731 (0.660, 0.810)	1.089 (0.949, 1.250)	1.070 (0.930, 1.232)	1.154 (0.997, 1.336)	0.950 (0.913, 0.988)	1.256 (1.087, 1.451)
DM + CKD	DM + IHD + CKD	365	0.980 (0.973, 0.987)	0.830 (0.715, 0.964)	1.570 (1.283, 1.920)	1.206 (0.982, 1.482)	1.236 (0.998, 1.531)	0.966 (0.912, 1.023)	1.017 (0.824, 1.255)
		730	0.982 (0.977, 0.988)	0.800 (0.711, 0.900)	1.506 (1.285, 1.765)	1.161 (0.987, 1.365)	1.347 (1.138, 1.594)	0.962 (0.919, 1.006)	1.026 (0.869, 1.211)
		1095	0.984 (0.979, 0.989)	0.813 (0.731, 0.904)	1.444 (1.252, 1.666)	1.158 (1.000, 1.340)	1.334 (1.146, 1.553)	0.947 (0.909, 0.986)	0.981 (0.845, 1.140)
		1460	0.985 (0.980, 0.990)	0.835 (0.752, 0.927)	1.450 (1.260, 1.669)	1.218 (1.055, 1.406)	1.404 (1.209, 1.630)	0.941 (0.904, 0.979)	0.979 (0.845, 1.134)
IHD + CKD	DM + IHD + CKD	365	1.005 (0.996, 1.014)	0.677 (0.558, 0.822)	1.367 (1.053, 1.775)	1.242 (0.951, 1.621)	1.296 (0.982, 1.711)	0.981 (0.911, 1.057)	1.307 (0.995, 1.717)
		730	1.008 (1.000, 1.015)	0.635 (0.543, 0.742)	1.277 (1.034, 1.576)	1.080 (0.871, 1.340)	1.273 (1.017, 1.593)	0.983 (0.926, 1.044)	1.408 (1.130, 1.756)
		1095	1.006 (0.999, 1.012)	0.678 (0.589, 0.780)	1.294 (1.071, 1.563)	1.049 (0.865, 1.272)	1.188 (0.972, 1.451)	0.980 (0.929, 1.034)	1.287 (1.057, 1.568)
		1460	1.006 (0.999, 1.012)	0.691 (0.602, 0.792)	1.242 (1.033, 1.493)	1.066 (0.883, 1.286)	1.172 (0.963, 1.426)	0.976 (0.926, 1.029)	1.230 (1.014, 1.491)
DM	Death	365	1.007 (1.003, 1.011)	0.879 (0.809, 0.956)	1.144 (1.022, 1.280)	1.074 (0.957, 1.204)	1.138 (1.010, 1.283)	1.131 (1.096, 1.168)	1.097 (0.975, 1.233)
		730	1.009 (1.004, 1.014)	0.895 (0.811, 0.988)	1.200 (1.050, 1.371)	1.016 (0.886, 1.163)	1.094 (0.950, 1.261)	1.136 (1.094, 1.180)	1.266 (1.102, 1.455)
		1095	1.011 (1.006, 1.017)	0.913 (0.819, 1.019)	1.209 (1.043, 1.401)	1.025 (0.881, 1.192)	1.120 (0.957, 1.310)	1.125 (1.079, 1.173)	1.319 (1.131, 1.540)
		1460	1.014 (1.008, 1.021)	0.949 (0.835, 1.080)	1.145 (0.963, 1.361)	0.966 (0.810, 1.153)	1.039 (0.865, 1.249)	1.091 (1.039, 1.146)	1.305 (1.089, 1.565)
IHD	Death	365	1.030 (1.026, 1.034)	0.880 (0.810, 0.957)	0.808 (0.722, 0.904)	0.745 (0.664, 0.835)	0.885 (0.786, 0.997)	1.204 (1.166, 1.243)	1.220 (1.085, 1.372)
		730	1.029 (1.025, 1.033)	0.885 (0.818, 0.958)	0.797 (0.716, 0.887)	0.731 (0.655, 0.815)	0.830 (0.741, 0.929)	1.195 (1.159, 1.232)	1.300 (1.163, 1.453)
		1095	1.030 (1.026, 1.034)	0.892 (0.825, 0.964)	0.819 (0.737, 0.910)	0.729 (0.655, 0.811)	0.807 (0.722, 0.902)	1.183 (1.148, 1.219)	1.381 (1.238, 1.541)
		1460	1.031 (1.027, 1.035)	0.902 (0.827, 0.982)	0.864 (0.770, 0.970)	0.758 (0.674, 0.853)	0.825 (0.729, 0.932)	1.161 (1.123, 1.200)	1.362 (1.207, 1.537)
CKD	Death	365	1.027 (1.024, 1.031)	0.866 (0.803, 0.933)	0.958 (0.866, 1.060)	0.916 (0.826, 1.015)	0.928 (0.833, 1.033)	1.341 (1.303, 1.380)	1.411 (1.269, 1.568)
		730	1.028 (1.025, 1.031)	0.872 (0.812, 0.936)	0.934 (0.849, 1.028)	0.851 (0.772, 0.937)	0.899 (0.813, 0.995)	1.313 (1.278, 1.349)	1.461 (1.323, 1.614)
		1095	1.030 (1.026, 1.034)	0.915 (0.852, 0.984)	0.931 (0.845, 1.026)	0.859 (0.778, 0.949)	0.920 (0.829, 1.020)	1.286 (1.251, 1.322)	1.473 (1.331, 1.631)
		1460	1.032 (1.028, 1.036)	0.932 (0.858, 1.013)	0.910 (0.813, 1.017)	0.915 (0.816, 1.026)	0.882 (0.783, 0.994)	1.243 (1.204, 1.283)	1.508 (1.341, 1.695)
DM + IHD	Death	365	1.018 (1.007, 1.030)	0.676 (0.530, 0.862)	1.107 (0.798, 1.536)	0.908 (0.650, 1.269)	1.015 (0.717, 1.438)	1.070 (0.974, 1.175)	1.053 (0.748, 1.484)
		730	1.018 (1.009, 1.027)	0.712 (0.588, 0.862)	1.094 (0.846, 1.415)	1.051 (0.808, 1.366)	1.012 (0.770, 1.330)	1.071 (0.995, 1.153)	1.051 (0.803, 1.376)
		1095	1.016 (1.008, 1.024)	0.742 (0.628, 0.877)	1.135 (0.907, 1.422)	1.048 (0.833, 1.319)	1.016 (0.800, 1.290)	1.065 (0.999, 1.135)	1.091 (0.863, 1.381)
		1460	1.018 (1.011, 1.025)	0.715 (0.614, 0.832)	1.066 (0.869, 1.308)	0.984 (0.798, 1.212)	1.020 (0.820, 1.267)	1.032 (0.973, 1.093)	1.299 (1.049, 1.608)
DM + CKD	Death	365	1.008 (0.999, 1.017)	0.814 (0.671, 0.986)	1.409 (1.087, 1.825)	1.209 (0.928, 1.575)	1.215 (0.922, 1.600)	1.110 (1.031, 1.195)	0.820 (0.626, 1.075)
		730	1.010 (1.003, 1.018)	0.833 (0.711, 0.975)	1.311 (1.061, 1.621)	1.166 (0.939, 1.448)	1.202 (0.959, 1.505)	1.085 (1.022, 1.153)	0.970 (0.777, 1.210)
		1095	1.009 (1.003, 1.016)	0.819 (0.713, 0.941)	1.293 (1.072, 1.559)	1.117 (0.922, 1.352)	1.165 (0.955, 1.422)	1.067 (1.012, 1.126)	0.970 (0.798, 1.180)
		1460	1.009 (1.003, 1.015)	0.834 (0.729, 0.953)	1.253 (1.047, 1.501)	1.158 (0.964, 1.392)	1.195 (0.987, 1.447)	1.050 (0.998, 1.106)	0.970 (0.803, 1.171)
IHD + CKD	Death	365	1.031 (1.026, 1.037)	0.862 (0.772, 0.962)	0.973 (0.839, 1.128)	0.837 (0.719, 0.973)	0.970 (0.829, 1.135)	1.166 (1.117, 1.216)	1.363 (1.167, 1.591)
		730	1.031 (1.026, 1.035)	0.840 (0.766, 0.921)	0.987 (0.872, 1.117)	0.850 (0.749, 0.965)	0.978 (0.857, 1.116)	1.149 (1.109, 1.190)	1.347 (1.183, 1.534)
		1095	1.031 (1.027, 1.035)	0.849 (0.782, 0.923)	0.977 (0.874, 1.093)	0.853 (0.761, 0.956)	0.973 (0.864, 1.096)	1.138 (1.102, 1.175)	1.328 (1.181, 1.492)
		1460	1.031 (1.027, 1.035)	0.840 (0.775, 0.911)	0.970 (0.869, 1.082)	0.901 (0.806, 1.007)	0.997 (0.888, 1.120)	1.131 (1.097, 1.167)	1.350 (1.204, 1.513)
DM + IHD + CKD	Death	365	1.015 (1.007, 1.023)	0.610 (0.515, 0.723)	1.474 (1.172, 1.853)	1.436 (1.136, 1.815)	1.544 (1.210, 1.970)	1.032 (0.968, 1.086)	1.196 (0.941, 1.520)
		730	1.013 (1.006, 1.020)	0.642 (0.558, 0.738)	1.402 (1.161, 1.692)	1.256 (1.036, 1.522)	1.433 (1.173, 1.751)	1.024 (0.974, 1.078)	1.189 (0.976, 1.447)
		1095	1.012 (1.006, 1.018)	0.688 (0.609, 0.777)	1.353 (1.148, 1.595)	1.202 (1.017, 1.422)	1.439 (1.209, 1.713)	1.027 (0.989, 1.067)	1.196 (1.007, 1.420)
		1460	1.011 (1.005, 1.016)	0.704 (0.628, 0.788)	1.443 (1.238, 1.682)	1.309 (1.119, 1.530)	1.481 (1.259, 1.744)	1.041 (0.995, 1.087)	1.202 (1.024, 1.411)

Age had a clear influence on the transition mechanism. Transitions that were directed towards Death state from IHD to IHD + CKD and from CKD to IHD + CKD showed significantly increased risks due to increase in age. However, age appeared to negatively affect the cumulative hazards of several transitions such as from DM to DM + CKD, from CKD to DM + CKD, and from DM + CKD to DM + IHD + CKD, showing significantly lower cumulative hazards for older subjects.

As one of the interesting outcomes of this study, we found that males had a higher risk for transitioning between states compared to females, considering all types of transitions in the system. Among 16 transitions types, such differences were found to be significant for 12 cases.

Racial/ethnic discrepancies were found to affect the cumulative transition hazards between states. Compared to Whites, Asians had higher risks for moving from DM to DM + CKD, from CKD to DM + CKD, from DM + CKD to DM + IHD + CKD, from IHD + CKD to DM + IHD + CKD, from DM + CKD to Death, and from DM + IHD + CKD to Death. However, the relative risk of transferring from IHD state to Death was significantly lower for Asians. For NHPIs, transition hazards from DM to DM + CKD, from IHD to DM + IHD, from CKD to DM + CKD, from DM + CKD to DM + IHD + CKD, and from DM + IHD + CKD to Death were relatively higher compared to Whites, but hazards from IHD to IHD + CKD and from IHD to Death were relatively lower for the NHPI group. The overall outcome of racial/ethnic differences seem to suggest that Whites had relatively lower risks for transferring from CKD to DM + CKD and from DM + IHD + CKD to Death, but they had an increased risk for transferring from IHD to Death compared to all other groups.

We observed that higher values of CCI were associated with increased risks of transitioning to the Death state. In several transitions such as from DM, IHD, CKD, and IHD + CKD states to Death, the increased risks were statistically significant. Furthermore, subjects with higher CCI were less likely to transition from DM to DM + IHD, from DM to DM + CKD, from IHD to DM + IHD, CKD to DMI + CKD, and from DM + CKD to DM + IHD + CKD.

Estimated relative risks for the dual eligibility suggested that it has a positive association with elderly subjects moving to the Death state. In fact, observed risks were significant for such transitions from all other states. Additionally, we found significantly increased risks associated with the dual eligibility on transitions from IHD to DM + IHD, from IHD to IHD + CKD, and from DM + IHD to DM + IHD + CKD.

## Discussion

We investigated characteristics of a multistate system that was based on states defined by three major chronic conditions, DM, IHD, and CKD, for elderly individuals aged 65 and above, using five years of Hawaii Medicare data. In the interconnected disease network, an individual entered the system from DM, IHD, or CKD states and finally reaches the absorbing state Death following one of several possible paths.

During the five-year follow-up, we found that the probabilities of occupying the initial states continuously decrease, showing tendencies of individuals moving to the subsequent states. In particular, an individual with CKD had only about a 50% chance to stay in this initial state at the end of the five-year study period, declining the occupational probability at a faster rate compared to DM and IHD states. This could possibly be due to the serious co-morbidity risks associated with CKD condition. During this period, the state occupational probabilities at intermediate states almost reached stationary levels, and the probability of death continuously increased following a fairly linear trend with respect to time. The analysis of marginal transition hazards provides a clear picture of burdens of competing hazards on transitioning to various subsequent states initiating from a given state. For example, subjects with IHD + CKD had a greater risk of death than their risk of moving to DM + IHD + CKD state, at any given time. On the other hand, subjects with DM + IHD had a higher risk of moving to DM + IHD + CKD state than the corresponding risk of death, at any given time.

The results revealed several interesting covariate relationships on state occupational probabilities and transition hazards. We found that females had a relatively lower hazard for transitioning between states compared to males. It is a known fact that females have a longer lifespan compared to males [20, 21]. Our results can possibly shed some light on this with respect to a chronic disease system. Among many racial/ethnic differences uncovered, we found that there was an increased risk for Asian and NHPI patients to transfer from DM to DM + CKD states while Whites had the highest risk of transferring from IHD to Death. Several other shared characteristics among Asians, NHPI, and Other groups compared to Whites were observed. Such racial/ethnic differences should be considered when healthcare providers/administrators develop early prevention strategies for subgroups of patients with higher risks.

We observed that older individuals were less likely to transfer from IHD to IHD + CKD, from CKD to DM + CKD, and from DM + CKD to DM + IHD + CKD. Also, individuals who had higher values of the CCI were less likely to transfer from several states to their subsequent states. Although some of these findings seem surprising, we want to draw attention to the magnitude of hazards by these factors on transferring directly to the Death state regardless of the magnitude of transitioning to subsequent clinical states, which were always high. We found that subjects with low economic status as reflected by their dual eligibility, had relatively high risks of death, which could possibly be due to living conditions, low income, or barriers to accessing healthcare.

Findings of this study can be strategically utilized and implemented in clinical and healthcare management settings to minimize mortality and transition risks among the elderly who are diagnosed with DM, IHD, and CKD conditions. We identified a wide range of covariate influences on state-to-state transition hazards. Subgroup-specific intervention plans can be established by considering the expected risks. For example, recommending periodic screenings or using tests with increased sensitivities in diagnosing diseases for high risk subgroups could provide a better chance for early detection or improved implementation of appropriate therapeutic strategies before those conditions worsen. High-risk subjects can be introduced to early prevention strategies or specialized care upon recommendations by their healthcare providers to minimize anticipated risks for comorbidities. Obviously, such approaches could result in extra cost. Hence, healthcare policymakers should evaluate these facts from a financial prospective, balancing the costs for required resources (including the patient care workforce for patients at more chronically ill stages) versus the cost of developing and implementing preventive strategies, as well as patient health and quality of life aspects.

There is a paucity of research literature exploring state-transition outcomes for chronic conditions including DM, IHD, and CKD within the general multistate framework. In contrast to ours, several studies utilizing multistate models focused on the progression of a given condition in different stages of the disease [22–26]. Other reports on DM, IHD, and CKD described risks of developing one condition given few other chronic conditions [27–31] and the burden of comorbidities among these patient populations [32–34]. In terms of survival, previous studies frequently focused on estimating hazards and mortality rates for DM, IHD, and CKD populations [35–37]. Interestingly, a study conducted using Third National Health and Nutritional Examination Survey revealed 10-year mortality rates as 7.7, 11.5, and 31.1% respectively for subjects without either diabetics or kidney disease, patients with diabetes but without kidney disease, and patients with both diabetes and kidney disease, demonstrating the devastating impact of co-occurring diabetic and kidney diseases [38]. This study also shed light on increased cardiovascular-related events rates for having both conditions together. Clearly, there is a lacking of research exploring the progressive patterns of DM, IHD, and CKD conditions and mortality in the multi-disease state-transition setting.

It is challenging and expensive to collect longitudinal data of a cohort of subjects that contains health status in a continuous time frame. However, health insurance databases are rich sources providing vital information which can be utilized in analyzing multistage disease transition systems similar to the model that we used. Compared to carefully collected follow-up data from a set of subjects for a few pre-selected conditions, health insurance data bases naturally provide access to a spectrum of diagnoses, which allows their widened applications to many public health problems.

Besides addressing the specific problem outlined based on diagnoses of DM, IHD, and CKD, a key interest of ours was to illustrate the use of health insurance data to evaluate survival characteristics of a chronic disease system. However, these approaches are not widely adopted yet. The large scale of the claims data, the intense effort needed to track subjects’ transitions among multiple clinical stages, and data security issues, are just several reasons for its lack of popularity. The use of health insurance claims data may not be an ideal choice to evaluate disease systems as these databases are incapable of updating the health status of the subject efficiently in real time. The system only documents when a subject makes a claim. Due to a variety of reasons, there can be a considerable number of unreported incidents even though the specific events of interest have already occurred, such as unawareness of the event, use of multiple insurance plans, switching between plans, delayed or irregular hospital visits, and errors in reporting, etc. These issues could lead to underestimation in state-to-state hazard estimations. However, it is quite reasonable to assume that these issues are independent from subject-specific covariates in the general sense. The generalizability of outcomes to a wider population is a common potential limitation for most of the studies conducted based on claims data, as the inferences made could be specific to the population under investigation. Nevertheless, we believe that the use of longitudinal health insurance data provides an excellent opportunity to investigate healthcare problems.

There are several other limitations with this study. We determined individual’s first disease occurrence history prior to 2009 using historical ICD-9 codes provided for several selected major conditions, but historical records of conditions can be limited and may not fully reflect the complete chronic conditions of the individual. This can possibly result in misidentification of subjects who had already diagnosed a chronic condition prior to 2009 as new cases, causing potential errors in survival estimates and in calculating the CCI. Furthermore, we found reporting errors exist in the claims data. We removed claims with dates found to be older than the date of death for several subjects. Although the proposed multistate model restricts individuals from entering into the system from intermediate states and transiting from initial states to the DM + IHD + CKD state, we observed such phenomenon for a handful of subjects in the data. However, to reduce the complexity, those beneficiaries were not included in the analysis. The maximum time period that these subjects were limited to was 5 years. To have a better understanding of such complex system, specifically in consideration of chronic diseases, a longer follow-up period is necessary. Due to the limited follow-up period the observed transitions subjected to right censoring. In fact, the right censoring rate in terms of an individual reaching to the final state was 87.2%. It is important to note that our analysis did not consider temporal effects associated with the calendar time. For instance, we assumed a given state-to-state transition probability is independent of the calendar time. However, such transitions could potentially be influenced by time-varying factors. It is worthwhile to conduct future sensitivity analyses to evaluate the robustness of the results due to the impact of these time varying factors. Lastly, several other potential risk factors may not be considered in our multistate model. For example, we did not include obesity that can be interrelated and contribute to the development and progression of the chronic conditions. We did not include obesity as a covariate because the identification of obesity using diagnosis codes tends to be highly underestimated.

We believe this is the first study conducted investigating DM, IHD, and CKD together in the prospect of state transitions and survival in the elderly population. The research revealed many important covariate relationships with respect to state occupational probabilities and transitions. The results will be useful for healthcare providers and policymakers in prevention planning and implementation.

## Conclusion

Using Hawaii Medicare database, we quantified survival characteristics of a multistate system that was defined by the occurrence of three chronic conditions (DM, IHD, and CKD) and their transition to the absorbing state Death, among individuals age 65 and above. Our study provides evidences that the clinical state transition mechanism could be subject- or subgroup-specific. Thus, consideration of subgroup-specific screening procedures and intervention plans for elderly individuals will be helpful for the optimal prevention and care of these chronic conditions.

## Additional file

Additional file 1: State occupation probabilities and transition hazards: A supplementary description of statistical methods used for estimating state occupation probabilities and transition hazards. **Table S1.** A summary matrix showing state-to-state transition counts for the chronic disease network. *NA* indicates transitions that are not applicable for the system. **Table S2.** Estimated regression coefficients that represent effects of covariates: age, gender, race/ethnicity, CCI and dual eligibility, on the state occupation probability at selected time points along with p-values (in brackets). **Table S3.** Estimated regression coefficients that represent effects of covariates: age, gender, race/ethnicity, CCI and dual eligibility, on cumulative transition hazards at selected time points along with p-values (in brackets). **Figure S1.** Marginally estimated cumulative state-to-state transition hazards from state DM to subsequent states. **Figure S2.** Marginally estimated cumulative state-to-state transition hazards from state IHD to subsequent states along with bootstrap based 95% point-wise confidence bands. **Figure S3.** Marginally estimated cumulative state-to-state transition hazards from state CKD to subsequent states along with bootstrap based 95% point-wise confidence bands. **Figure S4.** Marginally estimated cumulative state-to-state transition hazards from state DM+HD to subsequent states along with bootstrap based 95% point-wise confidence bands. **Figure S5.** Marginally estimated cumulative state-to-state transition hazards from state DM+CKD to subsequent states along with bootstrap based 95% point-wise confidence bands. **Figure S6.** Marginally estimated cumulative state-to-state transition hazards from state IHD+CKD to subsequent states along with bootstrap based 95% point-wise confidence bands. **Figure S7.** Marginally estimated state cumulative state-to-state transition hazards form state DM+IHD+CKD to Death state along with bootstrap based 95% point-wise confidence bands. (PDF 406 kb)

## Abbreviations

CCI: Charlson Comorbidity Index
CHD: Chronic Kidney Disease
DM: Diabetes mellitus
ICD-9: Classification of Disease 9th Revision
IHD: Ischemic Heart Disease
