# Supplementary material for: Progression of diabetes, ischemic heart disease, and chronic kidney disease in a three chronic conditions multistate model
Source: BMC Public Health. 2018 Jun 18;18:752. doi: 10.1186/s12889-018-5688-y (PMC6006736; doi:10.1186/s12889-018-5688-y)
Supplement: Supplementary file 1 — State occupation probabilities and transition hazards: A supplementary description of statistical methods used for estimating state occupation probabilities and transition hazards. Table S1. A summary matrix showing state-to-state transition counts for the chronic disease network. NA indicates transitions that are not applicable for the system. Table S2. Estimated regression coefficients that represent effects of covariates: age, gender, race/ethnicity, CCI and dual eligibility, on the state occupation probability at selected time points along with p-values (in brackets). Table S3. Estimated regression coefficients that represent effects of covariates: age, gender, race/ethnicity, CCI and dual eligibility, on cumulative transition hazards at selected time points along with p-values (in brackets). Figure S1. Marginally estimated cumulative state-to-state transition hazards from state DM to subsequent states. Figure S2. Marginally estimated cumulative state-to-state transition hazards from state IHD to subsequent states along with bootstrap based 95% point-wise confidence bands. Figure S3. Marginally estimated cumulative state-to-state transition hazards from state CKD to subsequent states along with bootstrap based 95% point-wise confidence bands. Figure S4. Marginally estimated cumulative state-to-state transition hazards from state DM+HD to subsequent states along with bootstrap based 95% point-wise confidence bands. Figure S5. Marginally estimated cumulative state-to-state transition hazards from state DM+CKD to subsequent states along with bootstrap based 95% point-wise confidence bands. Figure S6. Marginally estimated cumulative state-to-state transition hazards from state IHD+CKD to subsequent states along with bootstrap based 95% point-wise confidence bands. Figure S7. Marginally estimated state cumulative state-to-state transition hazards form state DM+IHD+CKD to Death state along with bootstrap based 95% point-wise confidence bands. (PDF 406 kb) [file 12889_2018_5688_MOESM1_ESM.pdf]

Supplementary Material

State occupation probabilities and transition hazards

To estimate marginal state occupational probabilities, we used the Aalen and Johnson estimator [16], which is a nonparametric technique that provides a great flexibility of handling a complex multistate system without using strict model assumptions. The estimation procedure of above quantities for a multistate model is formally described below.

Consider a time continuous multistate model with a finite state space $\mathscr{J} = \{1, .., J\}$, that allows a set of transitions among states. Note that, in the current problem, J is fixed to be eight. Suppose that a set of n individuals move independently in the multistate system. Consider the ith individual, $i = 1, ..., n$. Suppose $S_i(t)$ is the state occupies by the individual at time t. Let T_i^* be the time taken for the individual to reach the absorbing state and C_i be the right censoring time. Thus, $T_i = \min\{T_i^*, C_i\}$ is the observed time. Define $\delta_i = I(C_i \geq T_i^*)$ as the censoring indicator with respect to reaching the absorbing state for the ith individual. Suppose $X_i = (X_{i1}, .., X_{im})^T$ is a m dimensional covariate vector contains baseline information of the individual. Accordingly, data consist of independently and identically distributed copies of $\{S_i(u), 0 \leq u \leq T_i, \delta_i, X_i\}$, $1 \leq i \leq n$, for n subjects.

We estimate j to j' state-to-state transition counting process $N_{jj'}(t)$ by,

$$\hat{N}_{jj'}(t) = \sum_{i=1}^{n} I(C_i \geq t, S_i(t-) = j, S_i(t) = j'),$$

where $S_i(t-) = \lim_{u \to t-} S_i(u)$, is the state occupied just prior to time t. The estimate of the at-risk process of jth state $Y_j(t)$ is obtained by,

$$\hat{Y}_j(t) = \sum_{i=1}^{n} I(C_i \geq t, S_i(t-) = j).$$

Now, the Nelson-Aalen estimate of cumulative (integrated) state-to-state transition hazard $\hat{A}_{jj'}(t)$ can be computed by,

$$\hat{A}_{jj'}(t) = \begin{cases} \int_0^t Z(u)\hat{Y}_j(u)^{-1} d\hat{N}_{jj'}(u), & \text{if } j \neq j' \\ -\sum_{j \neq j'} \hat{A}_{jj'}(t), & \text{otherwise,} \end{cases}$$

with $Z(u) = I(\hat{Y}_j(u) > 0)$. The process given by $d\hat{N}_{jj'}(t)$ can be obtained by the corresponding jumps of state-to-state transitions within $[t-, t)$ window. This allows one to obtain an estimate of cumulative transition matrix of the system. Consider the

product integral

$$\hat{P}(s,t) = \prod_{(s,t]} (I_J + d\hat{A}),$$

where I_J is a $J \times J$ identity matrix. The estimator of jth state occupation probability, $j = 1, .., J$, $p_j(t) = Pr(S(t) = j)$ is given by,

$$\hat{p}_j(t) = \sum_{k=1}^{J} \frac{\hat{Y}_k(0+)}{n} \hat{p}_{kj}(0,t),$$

where $\hat{p}_{kj}(0,t)$ is the (k,j)th element of the matrix $\hat{P}(0,t)$. Note that the validity of such state occupation formulas even for non-Markov models have been established in the literature [22].

To identify the important covariates affecting on state occupation probabilities and state to state transition hazards, we employed a method developed using pseudo-values proposed by Anderson and Klein [17]. This uses a pseudo-values based regression approach starting with a marginal estimator, which could be both parametric or non-parametric, in order to take advantage of the flexible Generalized Estimating Equations approach of inference. The primary advantage of this approach is the interpretation of covariate effects. In fact, the method can be used for evaluating the direct effect of covariates on state occupation probabilities and cumulative transition hazards between states at a given time. Here pseudo-values of the state occupation probabilities are calculated using the jackknife estimator which yields,

$$\hat{p}_i^{ps}(t) = n\hat{p}_j(t) - (n-1)\hat{p}_j^{-i}(t), j = 1, .., J,$$

where $\hat{p}_j^{-i}(t)$ is the Aalen and Johansen [16] state occupation probability estimate at time t that obtained from a sample of size $n-1$ by eliminating the ith, individual from the data, and $\hat{p}_j(t)$ is the corresponding estimate calculated from the entire data. For the ith individual, the observed pseudo values for at a given time are dichotomized, which allows one to estimate a parametric regression model that relates state occupational indicators with baseline covariates. A similar approach was applied in estimating covariate effects on cumulative transition hazards, but without dichotomizing the estimated pseudo quantities. In this work, we used the generalized linear model with a *logit* link function to estimate the covariate effect on the state occupation, whereas *log* link function was used for state-to-state cumulative transition hazards.

Supplementary Tables

Table S1: A summary matrix showing state-to-state transition counts for the chronic disease network. *NA* indicates transitions that are not applicable for the system.

	To							
**From**	DM	IHD	CKD	DM+IHD	DM+CKD	IHD+CKD	DM+IHD+CKD	Death
DM	6,868	*NA*	*NA*	1,163	1,257	*NA*	*NA*	340
IHD		5,276	*NA*	1,008	*NA*	956	*NA*	703
CKD			3,295	*NA*	690	647	*NA*	727
DM+IHD				1,594	*NA*	*NA*	393	184
DM+CKD					1,348	*NA*	353	246
IHD+CKD						1,160	204	443
DM+IHD+CKD							587	363
Death								3,006

Table S2: Estimated regression coefficients that represent effects of covariates: age, gender, race/ethnicity, CCI and dual eligibility, on the state occupation probability at selected time points along with p-values (in brackets).

State	Days	Age	Gender (Female vs. Males)	Race/Ethnicity			CCI	Dual Eligibility
				Asian vs. White	NHPI vs. White	Others vs. White		
DM	0	-0.041 (<.001)	0.359 (<.001)	0.538 (<.001)	0.637 (<.001)	0.556 (<.001)	-0.247 (<.001)	-0.072 (0.066)
	365	-0.038 (<.001)	0.438 (<.001)	0.492 (<.001)	0.624 (<.001)	0.503 (<.001)	-0.282 (<.001)	-0.115 (0.005)
	730	-0.039 (<.001)	0.459 (<.001)	0.470 (<.001)	0.619 (<.001)	0.491 (<.001)	-0.285 (<.001)	-0.147 (<.001)
	1095	-0.039 (<.001)	0.472 (<.001)	0.452 (<.001)	0.597 (<.001)	0.473 (<.001)	-0.284 (<.001)	-0.151 (<.001)
	1460	-0.040 (<.001)	0.471 (<.001)	0.456 (<.001)	0.608 (<.001)	0.484 (<.001)	-0.281 (<.001)	-0.164 (0.000)
IHD	0	0.027 (<.001)	-0.182 (<.001)	-0.681 (<.001)	-0.558 (<.001)	-0.637 (<.001)	0.044 (<.001)	0.065 (0.110)
	365	0.018 (<.001)	-0.050 (0.101)	-0.685 (<.001)	-0.584 (<.001)	-0.694 (<.001)	0.003 (0.782)	-0.030 (0.495)
	730	0.015 (<.001)	-0.033 (0.292)	-0.684 (<.001)	-0.570 (<.001)	-0.677 (<.001)	-0.004 (0.732)	-0.077 (0.087)
	1095	0.013 (<.001)	-0.017 (0.580)	-0.695 (<.001)	-0.570 (<.001)	-0.684 (<.001)	-0.003 (0.796)	-0.119 (0.010)
	1460	0.011 (<.001)	-0.004 (0.898)	-0.696 (<.001)	-0.566 (<.001)	-0.695 (<.001)	-0.002 (0.861)	-0.130 (0.005)
CKD	0	0.023 (<.001)	-0.004 (0.911)	0.007 (0.860)	-0.145 (<.001)	-0.044 (0.340)	0.180 (<.001)	0.064 (0.150)
	365	0.019 (<.001)	0.119 (0.001)	-0.077 (0.098)	-0.184 (<.001)	-0.111 (0.028)	0.068 (<.001)	0.010 (0.842)
	730	0.016 (<.001)	0.142 (<.001)	-0.096 (0.049)	-0.175 (0.001)	-0.126 (0.017)	0.054 (<.001)	-0.019 (0.714)
	1095	0.015 (<.001)	0.126 (0.001)	-0.105 (0.034)	-0.165 (0.001)	-0.127 (0.018)	0.056 (<.001)	-0.017 (0.747)
	1460	0.013 (<.001)	0.129 (0.001)	-0.101 (0.044)	-0.180 (0.001)	-0.126 (0.020)	0.060 (<.001)	-0.042 (0.439)
DM+IHD	365	-0.009 (0.001)	-0.372 (<.001)	-0.059 (0.479)	0.154 (0.058)	0.108 (0.202)	-0.230 (<.001)	0.143 (0.083)
	730	-0.010 (<.001)	-0.307 (<.001)	-0.019 (0.798)	0.148 (0.044)	0.095 (0.219)	-0.222 (<.001)	0.064 (0.403)
	1095	-0.009 (<.001)	-0.297 (<.001)	-0.022 (0.760)	0.190 (0.008)	0.110 (0.142)	-0.227 (<.001)	0.096 (0.192)
	1460	-0.010 (<.001)	-0.249 (<.001)	-0.024 (0.740)	0.176 (0.013)	0.078 (0.299)	-0.233 (<.001)	0.059 (0.426)
DM+CKD	365	-0.026 (<.001)	-0.088 (0.166)	0.584 (<.001)	0.423 (<.001)	0.643 (<.001)	-0.190 (<.001)	-0.135 (0.133)
	730	-0.025 (<.001)	0.017 (0.773)	0.542 (<.001)	0.424 (<.001)	0.541 (<.001)	-0.211 (<.001)	-0.144 (0.083)
	1095	-0.025 (<.001)	0.066 (0.249)	0.552 (<.001)	0.446 (<.001)	0.557 (<.001)	-0.210 (<.001)	-0.142 (0.083)
	1460	-0.024 (<.001)	0.001 (0.981)	0.590 (<.001)	0.474 (<.001)	0.595 (<.001)	-0.225 (<.001)	-0.090 (0.248)
IHD+CKD	365	0.041 (<.001)	-0.425 (<.001)	-0.211 (0.028)	-0.318 (0.002)	-0.168 (0.113)	0.016 (0.536)	0.286 (0.004)
	730	0.037 (<.001)	-0.279 (<.001)	-0.277 (0.002)	-0.276 (0.003)	-0.234 (0.016)	0.007 (0.787)	0.212 (0.022)
	1095	0.033 (<.001)	-0.264 (<.001)	-0.281 (0.001)	-0.295 (0.001)	-0.205 (0.025)	0.003 (0.897)	0.125 (0.168)
	1460	0.033 (<.001)	-0.240 (<.001)	-0.296 (<.001)	-0.312 (<.001)	-0.203 (0.025)	-0.016 (0.515)	0.134 (0.136)
DM+IHD+CKD	365	-0.023 (<.001)	-0.667 (<.001)	0.579 (<.001)	0.378 (0.019)	0.403 (0.014)	-0.149 (0.019)	0.188 (0.191)
	730	-0.020 (<.001)	-0.639 (<.001)	0.548 (<.001)	0.275 (0.038)	0.616 (<.001)	-0.168 (<.001)	0.298 (0.007)
	1095	-0.021 (<.001)	-0.653 (<.001)	0.583 (<.001)	0.346 (0.004)	0.553 (<.001)	-0.242 (<.001)	0.159 (0.134)
	1460	-0.020 (<.001)	-0.646 (<.001)	0.510 (<.001)	0.313 (0.008)	0.620 (<.001)	-0.286 (<.001)	0.097 (0.363)
Death	365	0.078 (<.001)	-0.424 (<.001)	-0.140 (<.001)	-0.203 (0.012)	-0.070 (0.409)	0.390 (<.001)	0.395 (<.001)
	730	0.075 (<.001)	-0.459 (<.001)	-0.124 (<.001)	-0.292 (<.001)	-0.119 (0.098)	0.372 (<.001)	0.489 (<.001)
	1095	0.074 (<.001)	-0.419 (<.001)	-0.106 (<.001)	-0.280 (<.001)	-0.095 (0.152)	0.349 (<.001)	0.528 (<.001)
	1460	0.074 (<.001)	-0.426 (<.001)	-0.079 (<.001)	-0.216 (<.001)	-0.065 (0.309)	0.332 (<.001)	0.579 (<.001)

Table S3: Estimated regression coefficients that represent effects of covariates: age, gender, race/ethnicity, CCI and dual eligibility, on cumulative transition hazards at selected time points along with p-values (in brackets).

States		Days	Age	Gender (Females vs. Males)	Race/Ethnicity			CCI	Dual Eligibility
From	To				Asian vs. White	NHPI vs. White	Others vs. White		
DM	DM+IHD	365	-0.006 (0.001)	-0.147 (<.001)	-0.029 (0.579)	0.009 (0.875)	0.014 (0.806)	-0.062 (<.001)	0.168 (0.002)
		730	-0.004 (0.011)	-0.163 (<.001)	0.010 (0.840)	0.041 (0.402)	0.071 (0.161)	-0.066 (<.001)	0.129 (0.009)
		1095	-0.003 (0.051)	-0.164 (<.001)	0.053 (0.253)	0.115 (0.016)	0.105 (0.033)	-0.068 (<.001)	0.078 (0.106)
		1460	-0.001 (0.439)	-0.101 (0.006)	0.029 (0.558)	0.060 (0.226)	0.072 (0.169)	-0.076 (<.001)	0.085 (0.098)
DM	DM+CKD	365	-0.011 (<.001)	-0.058 (0.110)	0.229 (<.001)	0.145 (0.003)	0.243 (<.001)	-0.060 (<.001)	-0.026 (0.609)
		730	-0.010 (<.001)	-0.023 (0.493)	0.216 (<.001)	0.134 (0.003)	0.194 (<.001)	-0.064 (<.001)	-0.004 (0.930)
		1095	-0.009 (<.001)	-0.029 (0.384)	0.216 (<.001)	0.149 (0.001)	0.198 (<.001)	-0.060 (<.001)	-0.008 (0.855)
		1460	-0.008 (<.001)	-0.051 (0.148)	0.211 (<.001)	0.149 (0.002)	0.196 (<.001)	-0.054 (<.001)	0.042 (0.399)
IHD	DM+IHD	365	-0.004 (0.033)	-0.288 (<.001)	0.021 (0.697)	0.154 (0.005)	0.097 (0.086)	-0.045 (0.003)	0.062 (0.270)
		730	-0.005 (0.006)	-0.220 (<.001)	0.054 (0.271)	0.131 (0.009)	0.071 (0.178)	-0.040 (0.004)	0.049 (0.344)
		1095	-0.004 (0.023)	-0.217 (<.001)	0.073 (0.138)	0.132 (0.008)	0.045 (0.383)	-0.045 (0.001)	0.105 (0.040)
		1460	-0.003 (0.142)	-0.237 (<.001)	0.082 (0.109)	0.140 (0.007)	0.083 (0.124)	-0.052 (<.001)	0.105 (0.049)
IHD	IHD+CKD	365	0.022 (<.001)	-0.227 (<.001)	-0.031 (0.574)	-0.097 (0.083)	-0.001 (0.984)	0.054 (0.000)	0.210 (<.001)
		730	0.022 (<.001)	-0.208 (<.001)	-0.080 (0.116)	-0.124 (0.017)	-0.037 (0.491)	0.038 (0.008)	0.214 (<.001)
		1095	0.022 (<.001)	-0.207 (<.001)	-0.050 (0.317)	-0.117 (0.022)	0.004 (0.933)	0.028 (0.050)	0.153 (0.003)
		1460	0.022 (<.001)	-0.214 (<.001)	-0.081 (0.124)	-0.146 (0.007)	0.012 (0.826)	0.017 (0.246)	0.124 (0.025)
CKD	DM+CKD	365	-0.014 (<.001)	-0.132 (0.006)	0.351 (<.001)	0.196 (0.003)	0.339 (<.001)	-0.007 (0.685)	-0.029 (0.663)
		730	-0.011 (<.001)	-0.094 (0.035)	0.347 (<.001)	0.207 (0.001)	0.354 (<.001)	-0.017 (0.309)	-0.060 (0.337)
		1095	-0.011 (<.001)	-0.061 (0.172)	0.338 (<.001)	0.191 (0.002)	0.329 (<.001)	-0.036 (0.033)	-0.093 (0.139)
		1460	-0.010 (<.001)	-0.084 (0.066)	0.329 (<.001)	0.226 (<.001)	0.340 (<.001)	-0.039 (0.026)	-0.088 (0.169)
CKD	IHD+CKD	365	0.013 (<.001)	-0.184 (0.000)	-0.028 (0.675)	-0.129 (0.058)	-0.048 (0.504)	0.051 (0.008)	0.123 (0.078)
		730	0.013 (<.001)	-0.128 (0.003)	-0.013 (0.825)	-0.113 (0.060)	-0.057 (0.361)	0.038 (0.024)	0.096 (0.120)
		1095	0.013 (<.001)	-0.064 (0.137)	-0.010 (0.862)	-0.151 (0.011)	-0.086 (0.164)	0.037 (0.028)	0.045 (0.464)
		1460	0.014 (<.001)	-0.041 (0.368)	-0.037 (0.547)	-0.105 (0.093)	-0.066 (0.308)	0.023 (0.191)	0.105 (0.102)
DM+IHD	DM+IHD+CKD	365	0.001 (0.887)	-0.395 (<.001)	-0.081 (0.416)	-0.026 (0.799)	0.035 (0.741)	-0.031 (0.280)	0.303 (0.004)
		730	-0.002 (0.466)	-0.338 (<.001)	0.002 (0.982)	0.007 (0.932)	0.121 (0.157)	-0.040 (0.085)	0.303 (<.001)
		1095	-0.004 (0.168)	-0.305 (<.001)	0.075 (0.304)	0.065 (0.385)	0.128 (0.100)	-0.047 (0.025)	0.216 (0.005)
		1460	-0.001 (0.672)	-0.314 (<.001)	0.086 (0.223)	0.068 (0.345)	0.143 (0.055)	-0.052 (0.010)	0.228 (0.002)
DM+CKD	DM+IHD+CKD	365	-0.020 (<.001)	-0.186 (0.015)	0.451 (<.001)	0.187 (0.074)	0.212 (0.052)	-0.034 (0.239)	0.017 (0.877)
		730	-0.018 (<.001)	-0.223 (<.001)	0.409 (<.001)	0.149 (0.072)	0.298 (0.001)	-0.039 (0.092)	0.026 (0.763)
		1095	-0.016 (<.001)	-0.207 (<.001)	0.368 (<.001)	0.146 (0.050)	0.288 (<.001)	-0.055 (0.009)	-0.019 (0.804)
		1460	-0.015 (<.001)	-0.180 (0.001)	0.372 (<.001)	0.197 (0.007)	0.339 (0.000)	-0.061 (0.003)	-0.022 (0.774)
IHD+CKD	DM+IHD+CKD	365	0.005 (0.268)	-0.390 (<.001)	0.313 (0.019)	0.216 (0.112)	0.259 (0.067)	-0.019 (0.615)	0.268 (0.055)
		730	0.008 (0.047)	-0.455 (<.001)	0.244 (0.023)	0.077 (0.482)	0.241 (0.035)	-0.017 (0.583)	0.342 (0.002)
		1095	0.006 (0.087)	-0.388 (<.001)	0.258 (0.008)	0.048 (0.625)	0.172 (0.093)	-0.020 (0.467)	0.252 (0.012)
		1460	0.006 (0.094)	-0.370 (<.001)	0.217 (0.021)	0.064 (0.509)	0.159 (0.113)	-0.024 (0.365)	0.207 (0.036)
DM	Death	365	0.007 (0.001)	-0.129 (0.003)	0.134 (0.019)	0.071 (0.225)	0.130 (0.034)	0.123 (<.001)	0.092 (0.124)
		730	0.009 (<.001)	-0.111 (0.028)	0.182 (0.007)	0.015 (0.824)	0.090 (0.212)	0.128 (<.001)	0.236 (0.001)
		1095	0.011 (<.001)	-0.091 (0.106)	0.190 (0.012)	0.025 (0.749)	0.113 (0.158)	0.118 (<.001)	0.277 (<.001)
		1460	0.014 (<.001)	-0.052 (0.429)	0.135 (0.126)	-0.034 (0.703)	0.039 (0.682)	0.087 (0.001)	0.266 (0.004)
IHD	Death	365	0.030 (<.001)	-0.128 (0.003)	-0.214 (<.001)	-0.295 (<.001)	-0.122 (0.045)	0.186 (<.001)	0.199 (0.001)
		730	0.029 (<.001)	-0.122 (0.002)	-0.227 (<.001)	-0.314 (<.001)	-0.187 (0.001)	0.178 (<.001)	0.262 (<.001)
		1095	0.029 (<.001)	-0.115 (0.004)	-0.200 (<.001)	-0.316 (<.001)	-0.214 (0.000)	0.168 (<.001)	0.323 (<.001)
		1460	0.031 (<.001)	-0.104 (0.018)	-0.146 (0.013)	-0.277 (<.001)	-0.193 (0.002)	0.149 (<.001)	0.309 (<.001)
CKD	Death	365	0.027 (<.001)	-0.144 (<.001)	-0.043 (0.407)	-0.088 (0.094)	-0.075 (0.172)	0.293 (0.000)	0.344 (<.001)
		730	0.028 (<.001)	-0.137 (<.001)	-0.068 (0.161)	-0.162 (0.001)	-0.106 (0.039)	0.272 (0.000)	0.379 (<.001)
		1095	0.030 (<.001)	-0.088 (0.016)	-0.072 (0.148)	-0.152 (0.003)	-0.084 (0.112)	0.251 (0.000)	0.387 (<.001)
		1460	0.032 (<.001)	-0.070 (0.097)	-0.095 (0.097)	-0.089 (0.128)	-0.125 (0.039)	0.217 (0.000)	0.411 (<.001)
DM+IHD	Death	365	0.018 (0.002)	-0.392 (0.002)	0.102 (0.543)	-0.096 (0.572)	0.015 (0.932)	0.068 (0.156)	0.052 (0.766)
		730	0.018 (<.001)	-0.340 (<.001)	0.090 (0.494)	0.049 (0.713)	0.012 (0.934)	0.069 (0.066)	0.050 (0.715)
		1095	0.016 (<.001)	-0.299 (<.001)	0.127 (0.269)	0.047 (0.687)	0.015 (0.899)	0.063 (0.055)	0.088 (0.466)
		1460	0.018 (<.001)	-0.335 (<.001)	0.064 (0.540)	-0.016 (0.878)	0.019 (0.861)	0.031 (0.295)	0.261 (0.017)
DM+CKD	Death	365	0.008 (0.101)	-0.206 (0.036)	0.343 (0.010)	0.190 (0.160)	0.195 (0.166)	0.104 (0.006)	-0.198 (0.151)
		730	0.010 (0.008)	-0.183 (0.023)	0.271 (0.012)	0.154 (0.164)	0.184 (0.110)	0.082 (0.008)	-0.031 (0.786)
		1095	0.009 (0.005)	-0.200 (0.005)	0.257 (0.007)	0.110 (0.258)	0.153 (0.132)	0.065 (0.017)	-0.030 (0.763)
		1460	0.009 (0.005)	-0.182 (0.008)	0.226 (0.014)	0.147 (0.117)	0.178 (0.069)	0.049 (0.062)	-0.030 (0.752)
IHD+CKD	Death	365	0.031 (<.001)	-0.149 (0.008)	-0.027 (0.717)	-0.178 (0.021)	-0.030 (0.705)	0.153 (<.001)	0.309 (<.001)
		730	0.030 (<.001)	-0.174 (<.001)	-0.013 (0.835)	-0.163 (0.012)	-0.022 (0.739)	0.139 (<.001)	0.298 (<.001)
		1095	0.031 (<.001)	-0.163 (<.001)	-0.023 (0.686)	-0.159 (0.006)	-0.027 (0.651)	0.129 (<.001)	0.283 (<.001)
		1460	0.031 (<.001)	-0.174 (<.001)	-0.031 (0.581)	-0.104 (0.067)	-0.003 (0.958)	0.123 (<.001)	0.300 (<.001)
DM+IHD+CKD	Death	365	0.015 (<.001)	-0.494 (<.001)	0.388 (0.001)	0.362 (0.002)	0.434 (<.001)	0.034 (0.301)	0.179 (0.143)
		730	0.013 (<.001)	-0.444 (<.001)	0.338 (<.001)	0.228 (0.020)	0.360 (<.001)	0.022 (0.422)	0.173 (0.085)
		1095	0.012 (<.001)	-0.374 (<.001)	0.302 (<.001)	0.184 (0.031)	0.364 (<.001)	0.027 (0.256)	0.179 (0.041)
		1460	0.011 (<.001)	-0.352 (<.001)	0.366 (<.001)	0.269 (0.001)	0.393 (<.001)	0.037 (0.096)	0.184 (0.024)

Supplementary Figures

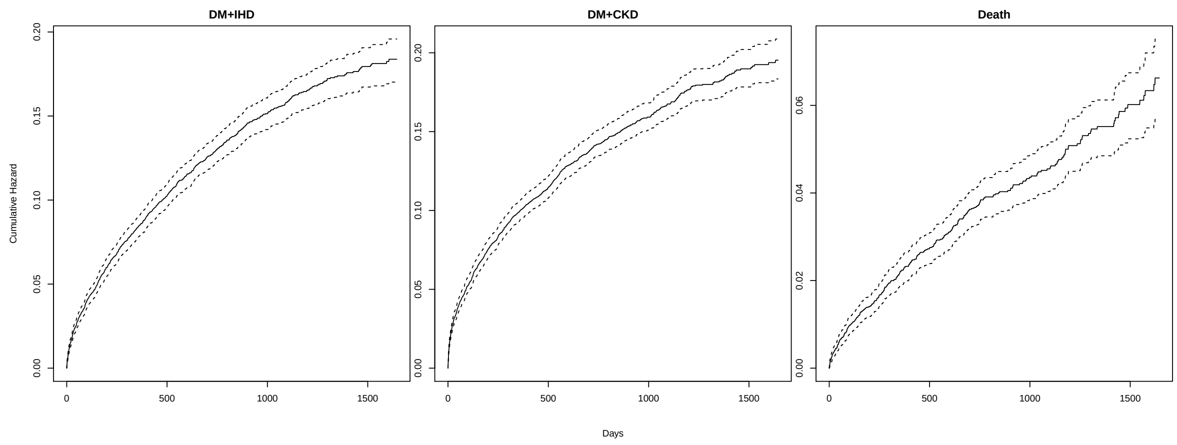

Figure S1: Marginally estimated cumulative state-to-state transition hazards from state DM to subsequent states.

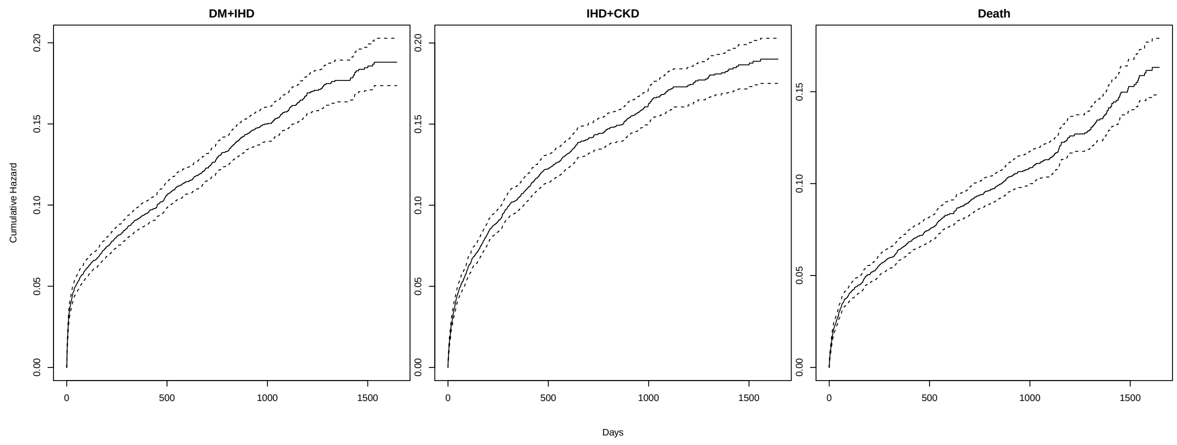

Figure S2: Marginally estimated cumulative state-to-state transition hazards from state IHD to subsequent states along with bootstrap based 95% point-wise confidence bands.

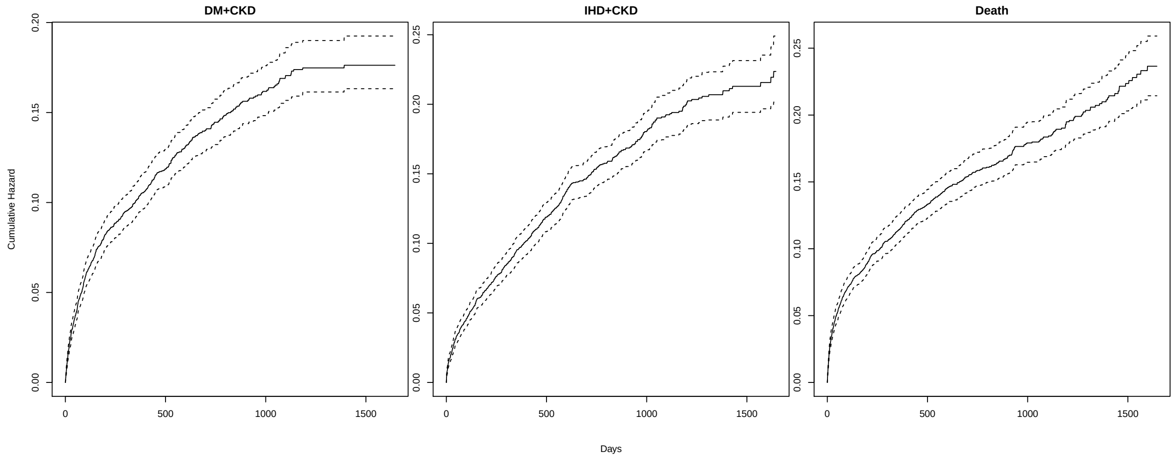

Figure S3: Marginally estimated cumulative state-to-state transition hazards from state CKD to subsequent states along with bootstrap based 95% point-wise confidence bands.

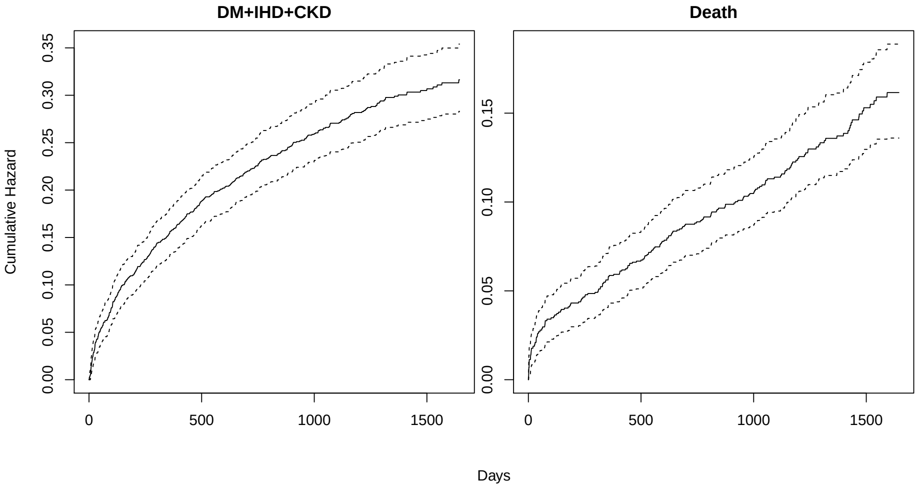

Figure S4: Marginally estimated cumulative state-to-state transition hazards from state DM+HD to subsequent states along with bootstrap based 95% point-wise confidence bands.

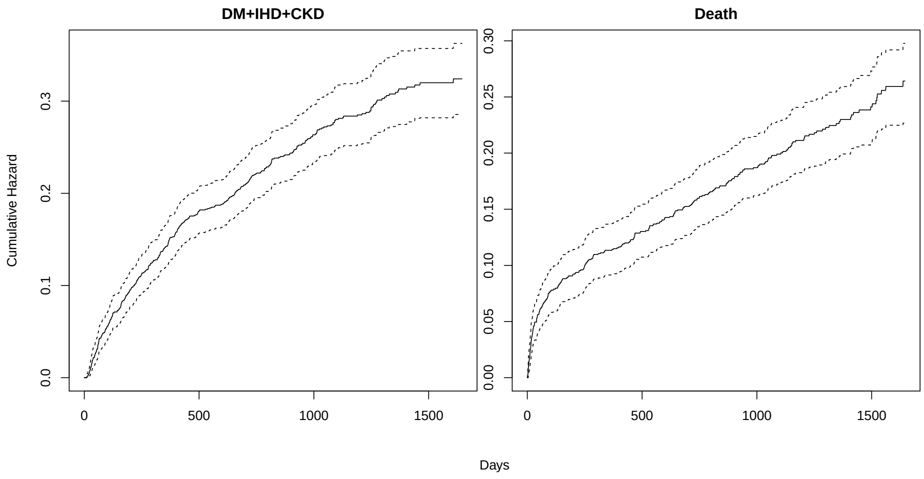

Figure S5: Marginally estimated cumulative state-to-state transition hazards from state DM+CKD to subsequent states along with bootstrap based 95% point-wise confidence bands.

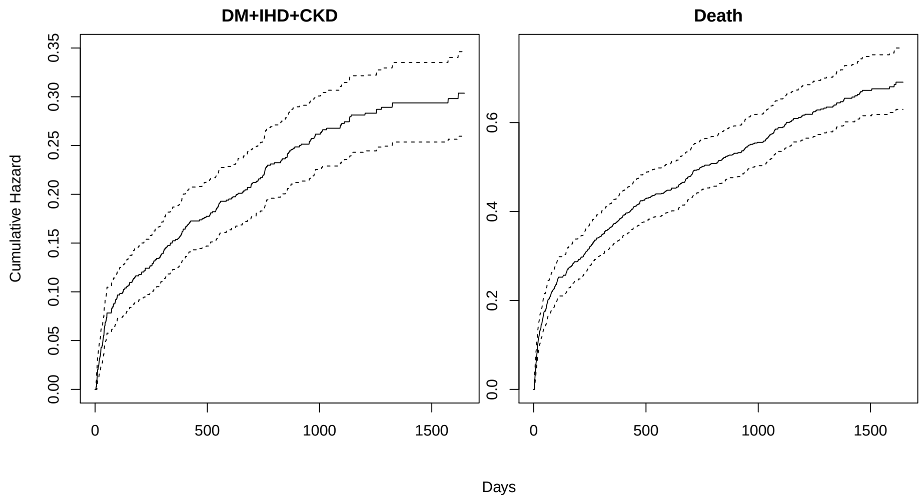

Figure S6: Marginally estimated cumulative state-to-state transition hazards from state IHD+CKD to subsequent states along with bootstrap based 95% point-wise confidence bands.

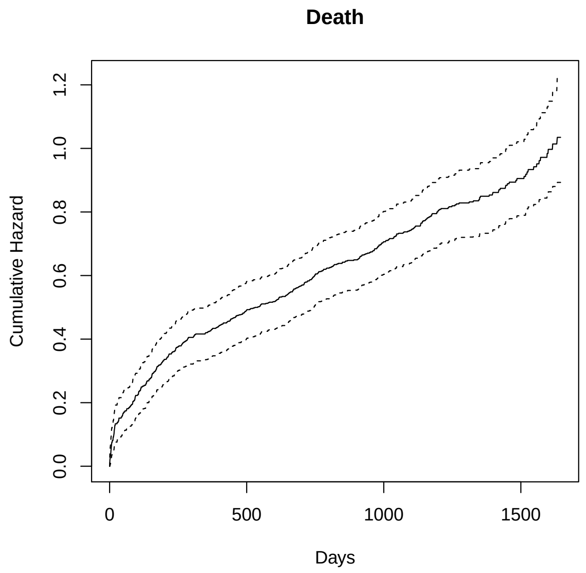

Figure S7: Marginally estimated state cumulative state-to-state transition hazards form state DM+IHD+CKD to Death state along with bootstrap based 95% point-wise confidence bands.
